# Editorial: Cancer burden, prevention and treatment in developing countries

**DOI:** 10.3389/fpubh.2022.1124473

**Published:** 2023-02-07

**Authors:** Junjie Huang, Paddy Ssentongo, Rajesh Sharma

**Affiliations:** ^1^The Jockey Club School of Public Health and Primary Care, Faculty of Medicine, The Chinese University of Hong Kong, Shatin, Hong Kong SAR, China; ^2^Centre for Health Education and Health Promotion, Faculty of Medicine, The Chinese University of Hong Kong, Shatin, Hong Kong SAR, China; ^3^Department of Medicine, Penn State Hershey Medical Center, Hershey, PA, United States; ^4^National Institute of Technology Kurukshetra, Kurukshetra, Haryana, India

**Keywords:** cancer prevention, developing countries, cancer burden, global health, cancer incidence and mortality

## Introduction

Cancer has become a global health issue of concern given the associated increased mortality and disability caused by it. In 2020, there were almost 18.1 million new cancer cases around the world (1). Early detection, cancer prevention, management and treatment have improved over the years in high-income countries with decreasing mortality and improved survival rates (2). However, the situation is different in low and middle-income countries (LMICs). With the rising population and exposure to risk factors, the number of new cancer incident cases has been rising significantly in LMICs in recent years (3). The survival rate of different cancers is lower in LMICs as they lack early detection programs, disease prevention, cost-effective treatment and oncologic infrastructure (4, 5). Early diagnosis of cancers is important for treatment options. However, LMICs lack access to early diagnosis, which results in higher mortality rates of several cancers in comparison with infectious diseases or malnutrition (5, 6). For example, effective low-cost screening methods for breast cancer are scarcely available in LMICs (6). Besides, the insufficient prevention, detection and treatment of infectious diseases also increases the risk of few cancers in the LMICs. Therefore, elucidating the burden and risk factors of cancers, which help in prevention and early detection is critical in LMICs to reduce cancer mortality and boost survival rates (7). In this special issue titled, “*Cancer burden, prevention and treatment in developing Countries*,” various facets of cancer burden were examined in the context of developing countries.

## Cancer burden in developing countries

Although cancer incidence and mortality rates are increasing in LMICs, there is a paucity of studies characterizing the geographic distribution and determinants of cancer among these countries. A recent study examined the cancer burden in Africa by the way of spatial epidemiology and also investigated the association between cancer burden and socioeconomic status of African countries (Sharma et al.). It has been found that almost 1.1 million new cases and 711,429 deaths occurred in 2020 because of cancers and human development index had a significant negative correlation with the mortality-to-incidence ratio (MIR). Furthermore, it was predicted that if the age-specific rates of cancers remain the same, then just because of rising population and changing demographics, the cancer burden will increase to 2.1 million new cases and 1.4 million deaths by 2040 in Africa. The study suggested that the cancer burden in African countries would increase even more than the predicted values by 2040 unless a holistic approach to cancer management and control is adopted in LMICs of Africa (Sharma et al.). Another study examining the burden of childhood cancers in 183 countries found that ~80% of the global burden of childhood cancers is experienced by the LMICs (8).

In Asia, the cancer incidence and mortality rates are also increasing. A recent study predicted the future mortality risk of breast cancer among East and South Asian (ESA) countries and identified the risk-attributable mortality caused by the breast cancer (Mubarik et al.). It has been found that the number of deaths caused by breast cancer will increase in the next 10 years in the ESA region. It was predicted that the age-standardized death rate of breast cancer will increase by 7.0% in East Asia, and 35.0% in South Asia from 1990 to 2030. Moreover, breast cancer mortality risk will increase in low-to-middle sociodemographic index countries in the region. It was suggested that early detection, cost-effective and timely treatment should be provided, and awareness must be increased to deal with rising breast cancer burden in ESA countries (Mubarik et al.). Another study examining the breast cancer burden in Asia found that due to late-disease presentation, lack of screening options and lower availability of treatment options, the mortality rates are higher and survival rates are lower in LMICs of Asia (5).

## Cancer prevention in developing countries

Previous research has identified that LMICs lacking early presentation and low cancer awareness along with poor oncologic infrastructure have low 5-year survival rates and high MIRs (9, 10). Some of the cancer burden in LMICs is also associated with infections. For example, in Nigeria, invasive cervical cancer (ICC) that causes serious public health burden is associated with the human immunodeficiency virus (HIV) (Musa et al.). A study investigated the relationship between ICC and epigenetic age acceleration by using cervical tissues of Nigerian women, who were diagnosed with ICC and HIV. They found an association between PhenoAge acceleration (PhenoAA) and ICC among women, who were infected with HIV. To improve the early detection of cancer based on the findings, considering resource constraints in LMICs, the study suggested that PhenoAA can be used as a potential biomarker to stratify the risk of HIV-associated ICC in Nigeria (Musa et al.).

Because the cancer control programs are not sufficient and comprehensive, LMICs are not able to deal with the increasing burden of cancer. Commonly, the countries are not able to allocate their budgets to implement comprehensive prevention strategies at primary, secondary, and tertiary levels (Akinyemiju et al.). Some of the suggested and discussed strategies to mitigate the high cancer burden in LMICs across different levels include encouraging routine cancer screening, creating awareness of modifiable risk factors including tobacco, alcohol, and vaccination, along with availability of comprehensive cancer treatment. Sustainable and comprehensive cancer control programs could be implemented by national plans and local communities to enhance cancer prevention and reduce the cancer burden in LMICs (11) (Akinyemiju et al.).

## Cancer treatment in developing countries

The major cancer treatment modalities in Africa are surgery, systemic therapies (hormonal therapy, targeted therapy, and chemotherapy), and radiotherapy, even these treatment strategies, are scarcely available in a timely manner and are associated with prohibitive cost (12, 13). A study explored the clinical and pathological factors that affected breast cancer mortality in the Eastern region of Ghana (Ssentongo et al.). The study investigated 129 patients and found that the 3-year survival rate was only 52%. It was due to late-stage presentation, insufficient capacity to diagnose accurately, analyze cancer subtypes, provide adequate treatment and follow-up (Ssentongo et al.). As suggested by this study, barriers and the possibility of improvements in breast cancer treatment and diagnosis based on sustainability and cost-effectiveness should be reviewed and identified in low-income countries (Ssentongo et al.).

Another study investigated cancer pain and treatment in French-speaking African countries using mixed methods (Hadjiat et al.). The study mentioned “the other opioid crisis” in developing countries, and stated the importance of understanding the context, nature and levels of cancer pain, and barriers to cancer treatment for effective pain management (Hadjiat et al.). The results of the study were collated into evaluative statements and recommendations in order to facilitate effective cancer pain treatment and management by considering the resources and conditions in the developing countries (Hadjiat et al.).

The accessibility of cancer treatment procedures and medicines is quite low in LMICs due to the high prices, low income, low health insurance and low public health spending (14) (Ocran Mattila et al.). After reviewing the pricing, affordability, availability, and accessibility of anti-cancer medicine in these countries, a study has found that cancer prices and availability varies widely across different countries and amongst different medicine brands (Ocran Mattila et al.). Patients at low-income levels can't afford the costly medicine and cancer treatments, thereby leading to therapy abandonment in LMICs (Ocran Mattila et al.). Therefore, medicine availability and prices, medicine-buying capacity of governments and related policies are important to ensure patients' accessibility to cancer medicine and treatment (Ocran Mattila et al.).

## Conclusion

Cancer mortality and morbidity is rising fastly in LMICs in the last few decades. Associated with socioeconomic status, the cancer burden in these countries is projected to increase, therefore, a holistic approach to cancer management must be carried out in LMICs, focusing on all aspects of cancer management and control such as prevention, cost-effective early detection and treatments. Cancer mortality and survival rates crucially depend upon early presentation, cancer awareness, and oncologic infrastructure, which can be improved by sustainable and comprehensive cancer control programs under the coordination of national plans and local communities. For cancer treatment and diagnosis, sustainability and cost-effectiveness are also important in facilitating effective cancer pain treatment and management by considering the resources and conditions of developing countries. Accessibility to cancer medicine either free or at affordable prices or universal health coverage of health expenditures must be ensured by the governments, particularly in the LMICs.

## Author contributions

JH prepared the first draft of the manuscript. RS and PS revised the manuscript. All authors approved the submission.
